# Assessing electron transfer reactions and catalysis in multicopper oxidases with operando X-ray absorption spectroscopy

**DOI:** 10.1038/s41467-019-14210-1

**Published:** 2020-01-16

**Authors:** Lucyano J. A. Macedo, Ayaz Hassan, Graziela C. Sedenho, Frank N. Crespilho

**Affiliations:** São Carlos Institute of Chemistry, University of São Paulo, São Paulo, 13560-970 Brazil

**Keywords:** Metalloproteins, Oxidoreductases, Electrocatalysis, Characterization and analytical techniques

## Abstract

Here we propose an experimental setup based on operando X-ray absorption spectroscopy (XAS) to understand why copper-containing oxidoreductase enzymes show exceptional performance as catalysts for the oxygen reduction reaction (ORR). An electrode based on carbon nanoparticles organized in mesoporous structures with bilirubin oxidase (BOD) was developed to be used in a home-made operando XAS electrochemical cell, and we probed the electron transfer under ORR regime. In the presence of molecular oxygen, the BOD cofactor containing 4 copper ions require an overpotential about 150 mV to be reduced as compared to that in the absence of oxygen. A second electron transfer step, which occurs faster than the cofactor reduction, suggests that the cooper ions act as a tridimensional redox active electronic bridges for the electron transfer reaction.

## Abstract (web summary)

Understanding enzyme active sites can elucidate fundamental enzymatic reaction pathways and inform designs for synthetic catalysts. Here, authors employ operando X-ray absorption spectroelectrochemistry to assess copper ions in bilirubin oxidase during oxygen reduction electrocatalysis.

## Introduction

**Fig. 1:**
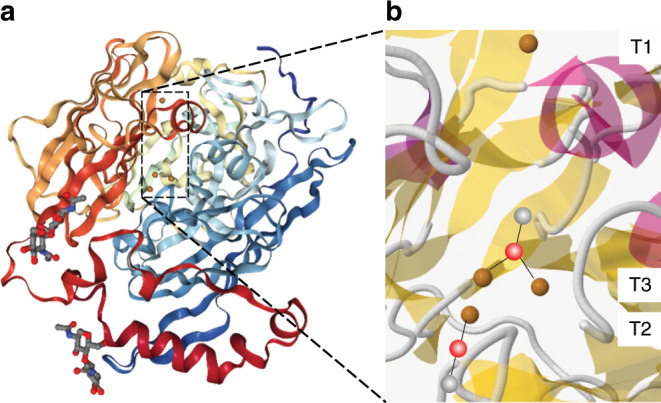
Structure of *Mv*BOD. **a** Representation of the crystal structure (PDB: 2XLL); **b** Zoom at the four Cu ions within *Mv*BOD in its resting oxidized state with the hydroxide bound to T3 and T2.

Metal ions are often present in biological molecules to provide easier routes for electron transfer so that the chemical reactions can proceed under physiological conditions^1^. In this context, multicopper oxidases (MCOs) are copper-containing oxidoreductase enzymes that show exceptional performance as catalysts for the oxygen reduction reaction (ORR) in a physiological environment, utilizing the electrons released from the oxidation of a particular organic substrate, for which the enzyme is designed^2^. More interestingly, the reaction pathway for the ORR, catalyzed by MCOs, leads to a 4-electron reduction to give water^3,4^. From an energy research perspective, there is a high demand for efficient catalysts for fuel^5,6^ and biofuel cells^7,8^ to facilitate the direct conversion of molecular oxygen (O_2_) to water. This will result in using the large amount of O_2_ in the atmosphere for clean energy conversion with the waste also being a clean chemical^9^. Some studies have proposed ways for the use of MCOs in cathodes for the ORR in biofuel cells, obtaining current densities up to several mA cm^−2^ (ref. ^10^). Bilirubin oxidases (BOD) belong to the group of MCOs that have 4 copper ions in its reactive site, divided into three groups, namely T1, T2, and T3 (Fig. 1). It is established that the trinuclear center (TNC) T2/T3 is the site mainly responsible for the electron transfer to O_2_, where it is further reduced to water^11,12^. Past studies have revealed the redox activity of the Cu ions by ex situ redox reactions and correlated this phenomenon with the electron transfer mechanism of the ORR^13^. Therefore, an in-depth analysis of the optimized catalysis, occurring in nature with the help of enzymes, might help in designing better and more robust catalysts and electrocatalysts for future technological applications^14,15^.

Herein, we investigate the pathway adopted by BOD that leads to the reduction of O_2_ to water, assessing the redox activity of the Cu ions in effecting the electron transfer from the substrate/electrode surface to the molecular oxygen. X-ray absorption spectroscopy (XAS) is a technique that allows the direct observation of not only a specific element, by using the internal transitions as a fingerprint, but also of the chemical environment surrounding it^11^. Therefore, we applied this technique to enzyme-modified carbon electrodes using a home-built sample holder as an electrochemical cell. The working electrode was placed in the focal plane of the X-ray beam in order to observe spectral changes of the Cu K-edge in the X-ray absorption near edge spectrum (XANES) region of the XAS spectrum, while the electrochemical potential on the enzymatic electrode was modulated and the catalytic process occurred. This helped to assess the redox behavior of the metallic ion cofactor and its role in the electron transfer mechanism of the metalloprotein.

## Results

### Bioelectrode construction

**Fig. 2:**
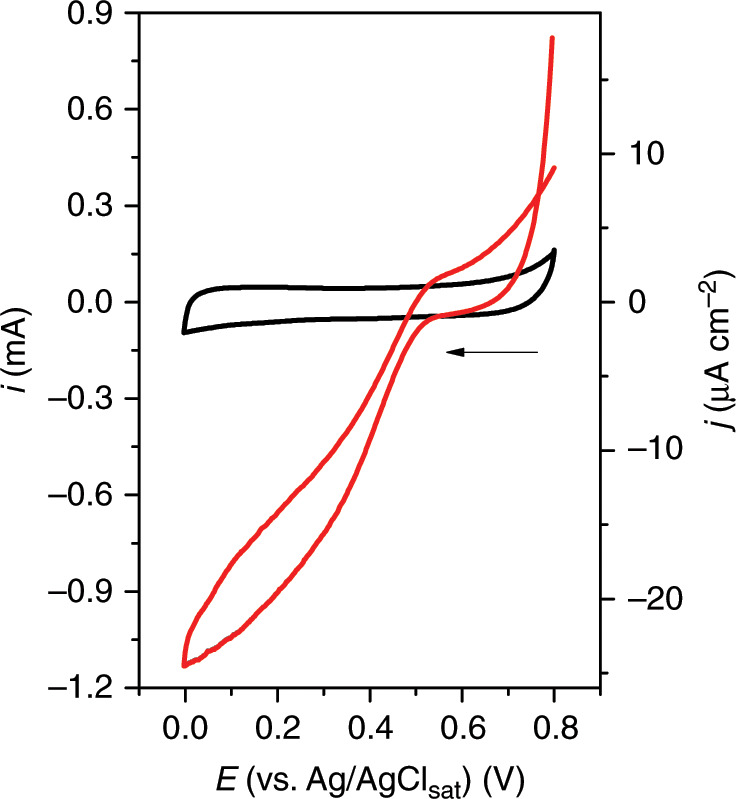
Electrochemical ORR by *Mv*BOD. Cyclic voltammogram of *Mv*BOD-modified electrode in an (red line) O_2_-saturated electrolyte performing the ORR and (black line) Ar saturated showing no bioelectrocatalytic process. Supporting electrolyte: 0.1 mol L^−1^ phosphate buffer (pH 7.2), *T* = 25 °C. Scan rate: 5 mV s^−1^.

Carbon cloth was used as a current collector platform for the working electrode modified by *Myrothecium verrucaria* BOD (*Mv*BOD). This carbon cloth underwent an oxidative treatment to generate oxygenated functional groups on its surface^16^, which have been attributed to the enhanced electron transfer properties between the redox species and the carbon electrodes^17^. A layer of carbon nanoparticles was also added onto the electrode surface to provide better electrochemical communication to the redox center of the enzyme due to its reduced size. These carbon nanoparticles create a mesoporous structure (Supplementary Fig. 1) on the surface that allows a more probable optimum conformation of the enzyme–electrode surface interaction^18^. The enzymes were then immobilized directly on this layer of carbon nanoparticles. Subsequently, the proton exchange polymeric membrane Nafion was used to trap the enzyme molecules and prevent them from dispersing in the electrolyte. Owing to the acidity of Nafion, we prepared the entrapment membrane in a buffered solution to avoid possible denaturation of the enzyme by local acidification. This possible alteration in the structure was checked by circular dichroism spectra of the enzyme and the enzyme with the buffered Nafion membrane and showed no significant changes in the spectrum thus confirming the integrity of the enzyme structure upon immobilization (Supplementary Fig. 2). The electrochemical ORR catalyzed by the *Mv*BOD-modified electrode is observed to occur, as per cyclic voltammogram (CV), with an onset potential of + 0.55 V (all potentials in this report are given with respect to Ag/AgCl_sat_) generating a catalytic current of approximately 1.1 mA or 24.7 µA cm^−2^ as we normalize by the electrochemical active surface area (ECSA) of the mesoporous surface on the electrode (Fig. 2). The electrocatalytic activity of this enzymatic electrode was corroborated using an oxygen-free electrolyte, by purging it with argon. No such electrocatalytic event is seen in this case, confirming the specificity and reversible activity of the enzyme, immobilized on the electrode, towards the ORR. As observed in Fig. 2, in addition to the O_2_ reduction process, an oxidative process around + 0.65 V can be seen. This oxidation process is also clearly seen in Supplementary Fig. 3 on the first cycle, and is much higher than the oxidative current obtained in the absence of enzyme. The origin of this process and its consequence on the redox process of the enzyme have been discussed in terms of the onset for water oxidation^19^. Although previous studies reported that the oxidation processes may be occurring within these oxidation currents^20^, water oxidation dominates the electrochemical process in the presence of BODs^19^. However, this does not disturb the background reduction currents, therefore obtaining an enzymatic electrode capable of performing ORR.

### *Operando* XAS results

**Fig. 3:**
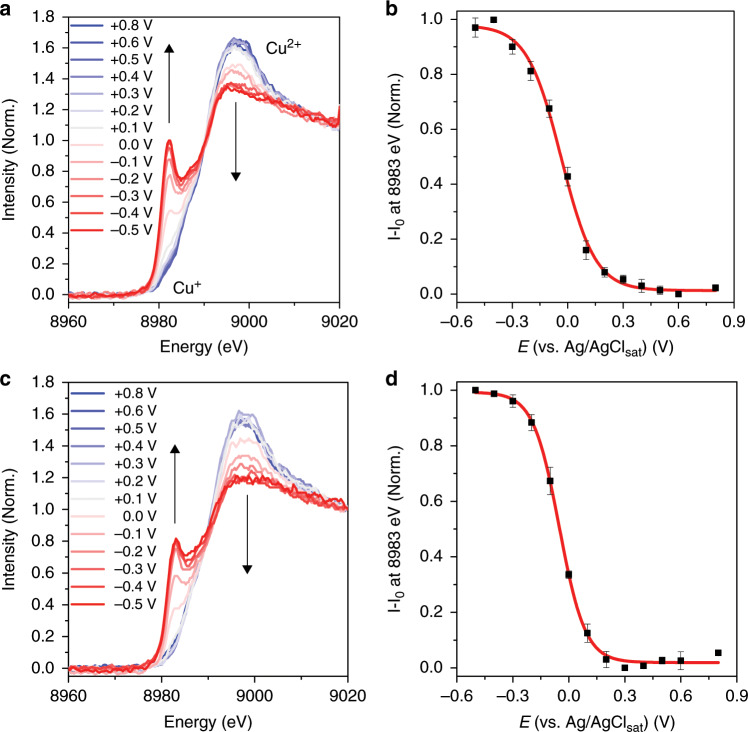
Cu K-edge XAS spectra under different conditions of electrochemical potential. **a** XAS spectra in the absence of oxygen. **b** Evolution of the signal at 8983 eV for reductive titrations in the absence of oxygen. **c** XAS spectra in the presence of oxygen. **d** Evolution of the signal at 8983 eV for reductive titrations in the presence of oxygen. Red lines in panels **b** and **d** represent the fits using the Nernst equation.

To obtain specific estimates of the catalytic activity, *operando* XAS spectroelectrochemical titration experiments were carried out by sweeping the potential in a window consisting of the resting condition and the energetically active enzymatic structure for the ORR, going towards more negative potentials to promote the reduction of the Cu ions. Firstly, we measured the Cu K-edge spectroscopic profile of the *Mv*BOD-modified electrode at the open circuit potential (OCP) (ca. + 0.54 V) to determine the resting state of the Cu ions in the enzyme. As previously reported by Solomon and co-workers^13^, MCOs, in their resting oxidized state, have all their Cu ions in the + 2 oxidation state. No signal specific to the + 1 oxidation state was observed at this potential in our case as well. Only an edge jump with a broad signal and a maximum at 8997 eV was observed, attributed to the 1 s → 4p transition of divalent Cu ions^11^ (Supplementary Fig. 4). We started the titration at + 0.8 V, where no catalytic current was obtained for the ORR, similar to what we observed in the CV. The XAS spectrum, at this high-applied potential, also shows the same features as that of the enzyme in its resting state. The edge jump, with maximum at 8997 eV (Fig. 3a, c), indicates that all Cu ions within *Mv*BOD are in the + 2 oxidation state under such electrochemical conditions.

**Fig. 4:**
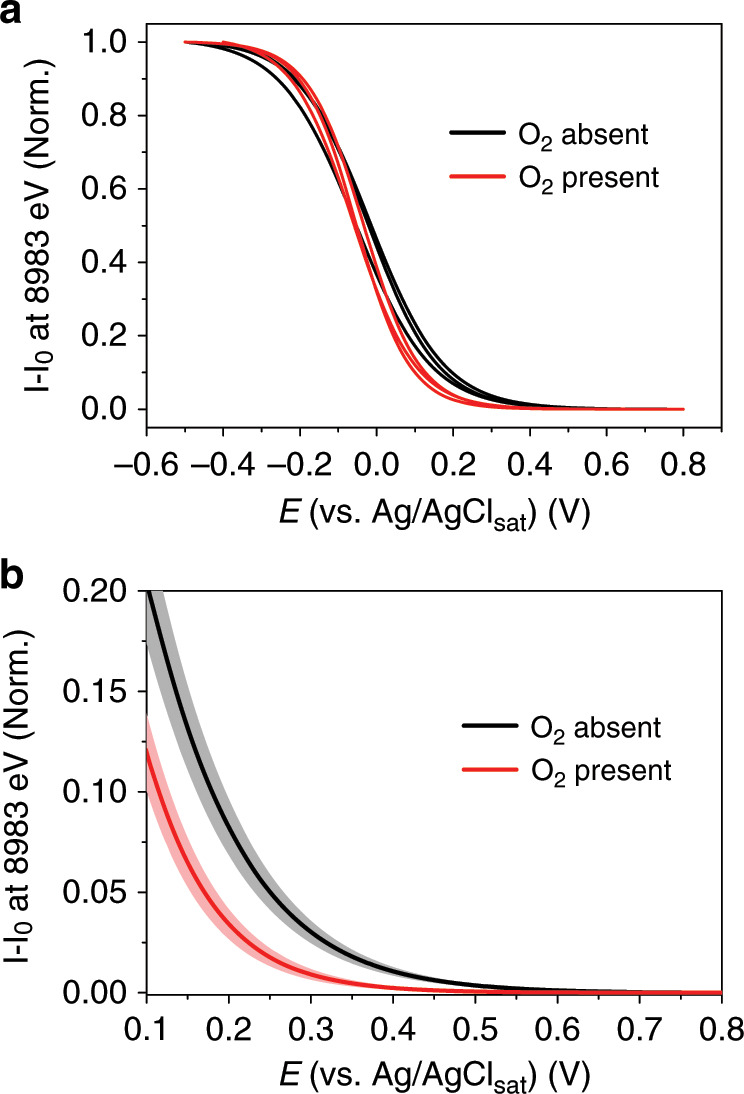
Cu^+^ potentiometric response in catalytic and non-catalytic conditions. **a** Cu K-edge potentiometric curves of Cu^+^ signal at 8983 eV (black line) in argon-saturated electrolyte and (red line) in oxygen-saturated electrolyte. **b** Highlighted region of the averaged curves in each condition where the rise in the signal at different overpotential values shows the shift between the curves (shades represent the standard deviation).

Under both experimental conditions, one where the electrolyte saturated with oxygen and the other where argon is used, the electrochemical activity of Cu ions within *Mv*BOD is eventually reached with the application of increasingly more negative potential, observed by the appearance of a signal at 8983 eV, attributed to the 1 s → 4p transition of monovalent Cu ions while the signal at 8997 eV is attenuated (Fig. 3a, c)^11^. This electrochemical reduction reaction of Cu^2+^ → Cu^+^ can be analyzed through the intensity evolution of the signal attributed to the Cu^+^ species at 8983 eV. One can still see an intense signal at 8997 eV even in the spectrum of the reduced *Mv*BOD. This signal is also characteristic to complexes having Cu^+^ as the coordination center^11^ but also could be possibly from enzyme molecules that did not respond to the electrochemical potential applied to the working electrode due to the large load of enzymes on the electrode surface. However, we are able to quantify and monitor the evolution of the signal emerging exclusively from the reactive species on the electrode by using subtractive characterization of resultant signals from the reduced species minus the initial state (*I* – *I*_0_). The evolution of the signal intensity at 8983 eV with respect to the potential sweep generates a sigmoidal shape curve characteristic to a Nernst-like behavior for a redox reaction (Fig. 3b, d). However, comparing the signal evolution for both experimental conditions, we observe a potential shift where the Cu ions start to become reduced (Fig. 4a). In contrast to the usual pattern for the electrochemical reduction of oxygen, no signal of Cu^+^ is observed in the potentiometric curve at low overpotential values close to + 0.55 V in the presence of oxygen. Looking closely at the signal intensities at 8983 eV in the fits (Fig. 4b), we observe that it only rises at approximately + 0.39 ± 0.01 V in this case, which means a 0.15 V overpotential is required to reduce and maintain the Cu ions in the + 1 oxidation state. In an oxygen-free environment, the potentiometric curves show a rise in the signal of the reduced Cu^+^ starting at + 0.55 V.

Interestingly, the shift in the absence of oxygen for the onset potential of the potentiometric curve that monitors the presence of Cu^+^ ions by the evolution of the XAS signal at 8983 eV (Fig. 4b, black line) towards a higher potential at + 0.53 ± 0.02 V coincides with the onset potential for the activity of the enzyme for the electrochemical ORR observed in the CV (Fig. 2, red line).

Fits presented in Fig. 3b, d (red lines) are obtained considering the redox reaction occurring as a single-step reaction, and were used exclusively to observe the shape of the curves and estimate the shift in the onset potential between them. For a more accurate fitting of the observed sigmoidal shaped titration curve, we took into account the possibility of four consecutive and cumulative 1-electron redox reactions that might have overlapped for the generation of the experimental curve, representing separately the reduction of each Cu ions (T1, T2, T3’, and T3”), algebraically expressed by Eq. (1):

1 $$I_{{\mathrm{total}}} = \, \left( {\frac{{I_{{\mathrm{max}}}^1}}{{\left\{ {1 + e^{\left[ {\left( {E - E_1^0} \right) \cdot \frac{{RT}}{{nF}}} \right]}} \right\}}}} \right) + \left( {\frac{{I_{{\mathrm{max}}}^2}}{{\left\{ {1 + e^{\left[ {\left( {E - E_2^0} \right) \cdot \frac{{RT}}{{nF}}} \right]}} \right\}}}} \right) \\ + \left( {\frac{{I_{{\mathrm{max}}}^3}}{{\left\{ {1 + e^{\left[ {\left( {E - E_3^0} \right) \cdot \frac{{RT}}{{nF}}} \right]}} \right\}}}} \right) + \left( {\frac{{I_{{\mathrm{max}}}^4}}{{\left\{ {1 + {\mathrm{e}}^{\left[ {\left( {E - E_4^0} \right) \cdot \frac{{RT}}{{nF}}} \right]}} \right\}}}} \right)$$

**Fig. 5:**
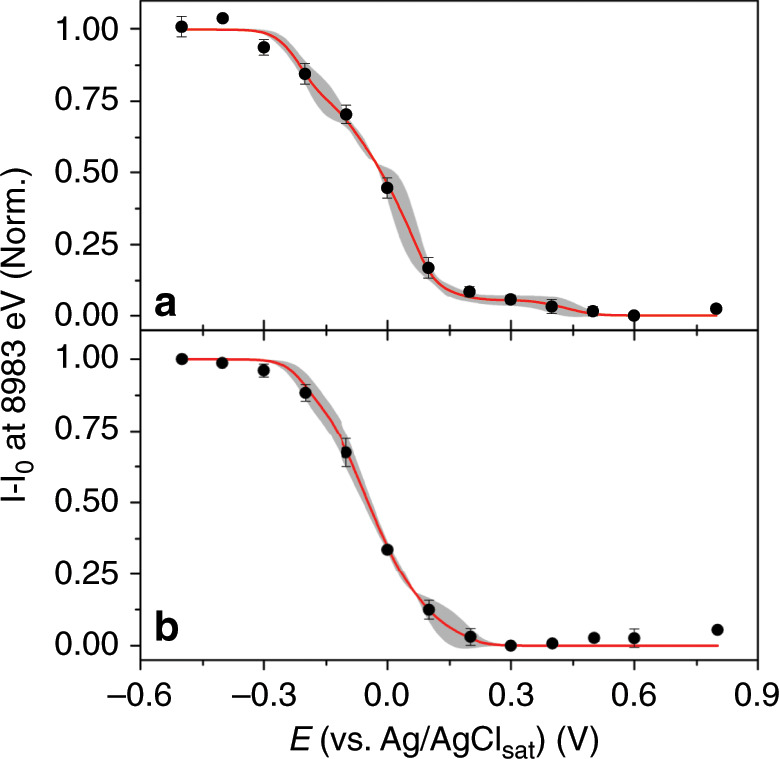
Obtaining information from individual and consecutive redox reactions. Titration curves for the experiments (**a**) in the absence of oxygen and (**b**) in the absence of oxygen. Dots represent the experimental data points; red lines are the fitted curve for the cumulative four 1-electron reduction; and gray shades represent the area-filled error obtained between the fits.

Where, *I*_total_ is the cumulative XAS signal intensity for all individual contributions from each redox reaction; ${\mathrm{I}}_{{\mathrm{max}}}^{{\mathrm{index}}}$ is the maximum XAS signal intensity of the sigmoidal curve from each individual redox reaction at a certain applied potential *E*; $E_{{\mathrm{index}}}^0$ is the midpoint potential of each individual redox reaction, in this case, the formal redox potential of each Cu center; *R* is the universal gas constant; *T* is the temperature at which the redox reaction is occurring; *n* is the number of electrons involved in the redox reaction; and *F* is the Faraday constant. Attempts to fit the experimental data considering these four overlapping individual titration curves all occurring by 1-electron reduction, we obtain an also sigmoidal shape curves that match more closely to the data points and minimizing the difference in slope between the curves (Fig. 5). Additionally to the shift in the onset potential for the starting of the electrochemical reduction of the Cu ions, in this approach we can estimate the formal potentials attributed to the reduction of each of these centers (Table 1). Interestingly, the most influenced center is the T1, whose midpoint potential at + 0.43 V downshifts to + 0.13 V. Even though the molecular oxygen does not bind to this center specifically, this reduction potential shift indicates the fast redox communication between the four Cu centers in the active site of *Mv*BOD.

**Table 1:** Midpoint potential of the Cu centers of *Mv*BOD for their consecutive 1-electron reduction.

	Midpoint potential (*E*^0^/*V* vs. Ag/AgCl_sat_)	
Cu center	Catalytic condition (oxygen available)	Non-catalytic condition (oxygen absent)
T1	+0.127 ± 0.061	+0.427 ± 0.034
T3’	−0.018 ± 0.029	+0.119 ± 0.056
T3”	−0.098 ± 0.021	−0.019 ± 0.034
T2	−0.197 ± 0.015	−0.185 ± 0.031

**Fig. 6:**
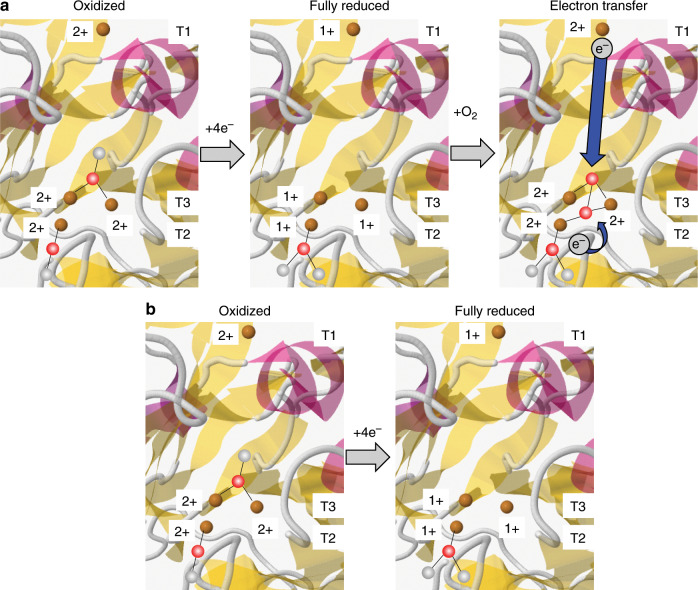
Simplified and schematic mechanism of internal electron transfer in *Mv*BOD. **a** In the presence and **b** absence of O_2_ bonded to the active site of *Mv*BOD.

The catalytic site of MCOs is characterized by the variation of oxidation state of the Cu ions and the modification of their coordination sphere, mainly related to the oxygenated species that is bound to the Cu centers (e.g., dioxygen, peroxide, and hydroxide) in this latter case^13^. The observation of shifts in the onset potential of the Cu centers to higher overpotentials suggest that although the Cu^2+^ ions within *Mv*BOD get reduced at a potential close to + 0.55 V during its electrocatalytic activity for ORR, they do not remain in the reduced oxidation state of + 1 due to the presence of oxygen in the reactive site, where the electrons are quickly transferred in a second reaction step to the O_2_ coordinated to the TNC T2/T3, hence returning to the + 2 oxidation state as schematically represented in Fig. 6a. In fact, when oxygen is not present in the TNC T2/T3, these electrons are not transferred, and thus Cu^+^ becomes the final electron acceptor and do not change back to the oxidized + 2 state (Fig. 6b). In order to promote the reduction of the Cu ions when oxygen is available to bind to the T2/T3 complex, a 0.15 V overpotential needs to be applied to the system. This provides the energy necessary to reduce O_2_, as well as to maintain Cu in the reduced state.

Usually, an internal electron transfer reaction using multi-step pathways is preferred to promote fast and efficient reactions even for long distance transfer mechanisms between the donor and the final electron acceptor. In this case, an electronic bridge can play an important role to minimize the time as well as the energy required to promote such a reaction, represented as a donor–bridge–acceptor (D–B–A) system^21,22^. Thus, acting more than an electronic shell that helps with the docking and reduction of O_2_, the catalytic center with the four Cu ions have to get reduced to the + 1 oxidation state to promote the effective electron transfer between the organic substrate (or electrode) and the molecular oxygen, behaving as a redox active bridge in a D–B–A system^23^.

## Discussion

In this work, we studied the fundamentals of the enzyme *Mv*BOD catalytic activity once it is immobilized on an electrode surface. Using an *operando* approach through the combination of XAS and electrochemical analysis provided a critical piece of information about the redox activity of the Cu ions within the enzymatic structure of *Mv*BOD and shed light on its role in the bioelectrochemical ORR catalyzed by MCOs. We were able to probe the behavior of the enzyme under specific conditions by monitoring the spectroscopic changes with respect to the changes in its energy state by the application of different electrochemical potentials to the enzyme-modified electrode, which showed the reaction of the metallic centers by the attenuation of the signal at 8997 eV attributed to the Cu^2+^ and rising of a signal at 8983 eV attributed to the Cu^+^ species on the enzymatic electrode. However, there is a higher overpotential needed to have the Cu^+^ ions when the ORR is happening, which does not happen when no oxygen is available to bind.

Additionally, the reduction of the metallic cofactors and a shift in the onset potential as well as in the midpoint potential for the effective reduction of the Cu ions, obtained by the separation of individual contributions from the reduction of each Cu center in the resultant titration curve, suggest their role as redox-active electronic bridges in the electron transfer mechanism promoting the reduction of dioxygen in the catalytic center of MCOs. This strategy of using the potentiometric titration in situ with XAS also opens up an alternative for quantifying the redox-active metal content within a biomolecule as the stoichiometry of electrons involved in the reaction also can be quantified. The 0.15 V shift, required for the reduction of the Cu ions under catalytic conditions points towards the stabilization of the TNC by dioxygen bonding, in which case more energy is needed for the reduction of both Cu ions and O_2_ as compared to that under the oxygen-free conditions.

In summary, these results investigating the ORR catalyzed by the enzyme *Mv*BOD demonstrate that the redox activity of metallic atoms within biomolecules can be directly assessed, gathering information about how the change in their oxidation state is able to promote the occurrence of the reaction under physiological environment. Such *operando* approach may provide deeper understanding on the mechanisms that metalloenzymes adopt in the catalytic processes that they are responsible for, mainly those that depend on electron transfer processes involving the metallic cofactor.

## Methods

### Reagents

NaH_2_PO_4_, Na_2_HPO_4_, H_2_SO_4_, and KMnO_4_ were obtained from Synth. Bilirubin oxidase from *Myrothecium verrucaria* (the cofactor structure was probed by electron paramagnetic resonance spectroscopy presented in Supplementary Fig. 5), isopropanol, and carbon nanoparticles were obtained from Sigma-Aldrich and used without further purification. All solutions were prepared with deionized water (18 MΩ cm at 25 °C).

### Electrode preparation

Carbon cloth (PWB-3) was obtained from Stackpole Electronics, Inc. Prior to its use as current collector, its surface was modified by reacting with KMnO_4_ and H_2_SO_4_ under sonication for 3 h as reported elsewhere^24^. On square pieces of this oxidized carbon cloth (CCo), 1 mg cm^−2^ of carbon nanoparticles previously dispersed in isopropanol were deposited. The immobilization of *Mv*BOD was performed by applying 50 µL of a solution containing 100 mg mL^−1^ of the enzyme in 0.1 mol L^−1^ phosphate buffer (pH 7.2) and drying under vacuum for 30 min. Subsequently, a layer of buffered Nafion salt was made to entrap the enzymes on the electrode by applying 30 µL of a 2.5 wt.% Nafion 117 in 0.1 mol L^−1^ phosphate buffer (pH 7.2) and also drying for 30 min under vacuum. The electrodes were kept at low temperature (4 °C) until their use.

### Electrochemical characterizations

A standard three-electrode electrochemical cell was employed for the study of the electrochemical profile of the modified *Mv*BOD-modified carbon electrode, this latter being used as working electrode while a platinum wire and Ag/AgCl_sat_ (in sat. KCl) were used as auxiliary and reference electrodes, respectively. All potentials reported here are referred to Ag/AgCl_sat_. These electrodes were controlled by an Autolab PGSTAT204 potentiostat/galvanostat.

Cyclic voltammetry was performed under constant gas flow for either purging the electrolyte with argon to remove oxygen or with oxygen to saturate the electrolyte for ORR. Conditions were set as stated in the figures captions. The electroactive area of the electrode was determined through a methodology taking into consideration the capacitive current of the electrode^25^. The specific capacitive current of the electrode was used to calculate the electrochemical active surface area (ECSA) of nanostructured materials. This method is based on the difference between the anodic (*i*_a_) and cathodic (*i*_c_) capacitive currents (Δ*i* = *i*_a _– *i*_c_) obtained under different scan rates where no faradaic processes occur (Supplementary Fig. 6a). The slope of the dependence of the Δi as function of the scan rate is related to the double layer capacitance (*C*_dl_) (Supplementary Fig. 6b) and calculated as:

2 $$C_{{\mathrm{dl}}} = \frac{1}{2}\cdot \frac{{\partial (\Delta i)}}{{\partial ({\mathrm{scan}}\;{\mathrm{rate}})}}$$

As the ECSA is related to the specific capacitance (Cs), which for carbon is 25 µF cm^−2^:

3 $${\mathrm{ECSA}} = \frac{{C_{{\mathrm{dl}}}}}{{C_s}}$$

we obtain an ECSA of the electrode equals to 46 cm^2^.

### *Operando* XAS measurements

Cu K-edge XAS spectra measurements were performed at the XAFS2 beamline of the Brazilian Synchrotron Light Laboratory (LNLS) in the fluorescence mode (Supplementary Fig. 7). A home-made spectroelectrochemical cell was utilized for this purpose. A Canberra 15-element solid-state Ge detector was used to collect Cu K-α fluorescence. In the focal plane, we placed the *Mv*BOD-modified carbon working electrode in direct contact with a Kapton tape window to minimize the interference from the electrolyte. Simultaneously to the sample spectrum, spectra of a Cu foil was used as reference and the calibration was performed using a Cu foil as internal reference, whose zero-crossing point of the second derivative spectrum was set as 8979 eV. Prior to the spectroscopic measurement, we scanned the electrode in order to find the highest fluorescence signal from Cu atoms and set fix that spot for all subsequent measurements as the focal point of the X-ray beam analyzes a region with 450 µm in diameter.

The electrochemical section of XAS experiment was composed of the *Mv*BOD-modified carbon as working electrode, Pt wire as counter-electrode, and Ag/AgCl_sat_ (in sat. KCl) as reference electrode. These electrodes were controlled by an Autolab PGSTAT204 potentiostat/galvanostat. In each spectrum, the potential was set fix 10 min before starting the measurement to equilibrate the protein film on the electrode, as well as its interface and kept fix until the end of the spectral scan.

These measurements were performed for three different electrodes, where all the working electrodes underwent spectroscopic measurements in each of the potentials ranging from + 0.8 to –0.5 V in both experimental conditions with the electrolyte saturated with either argon or oxygen. The order that the experiments were conducted was followed for all electrodes consisting in first measuring in an oxygen-free and subsequently in an oxygen-saturated electrolyte.

The enzyme stability for beam damage was checked by collecting spectra of the same spot of the electrode at the first measurement and after 6 h of irradiation (Supplementary Fig. 8).

## Supplementary information

Supplementary Material

## Data Availability

The data that supports the findings in the current study are available from the corresponding author upon reasonable request.
