# Supplementary material for: Assessing electron transfer reactions and catalysis in multicopper oxidases with operando X-ray absorption spectroscopy
Source: Nat Commun. 2020 Jan 16;11:316. doi: 10.1038/s41467-019-14210-1 (PMC6965173; doi:10.1038/s41467-019-14210-1)
Supplement: Supplementary file 1 — Supplementary Material [file 41467_2019_14210_MOESM1_ESM.pdf]

Supplementary Material

Assessing Electron Transfer Reactions and Catalysis in Multicopper Oxidases with *Operando* X-ray Absorption Spectroscopy

*Lucyano J. A. Macedo, Ayaz Hassan, Graziela C. Sedenho, and Frank N. Crespilho**

São Carlos Institute of Chemistry, Univeristy of São Paulo, São Carlos-SP 13560-970, Brazil
*frankcrespilho@iqsc.usp.br

Supplementary Figures

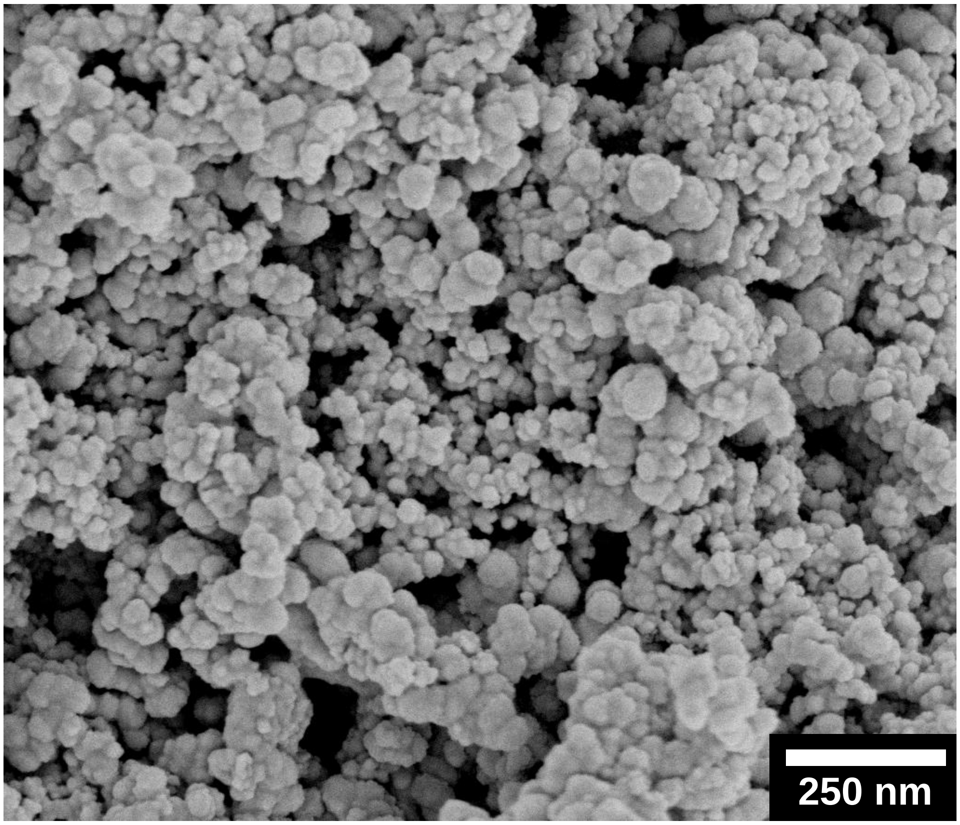

Supplementary Figure 1. Morphology of the electrode surface. SEM image of the carbon nanoparticles used on the working electrode.

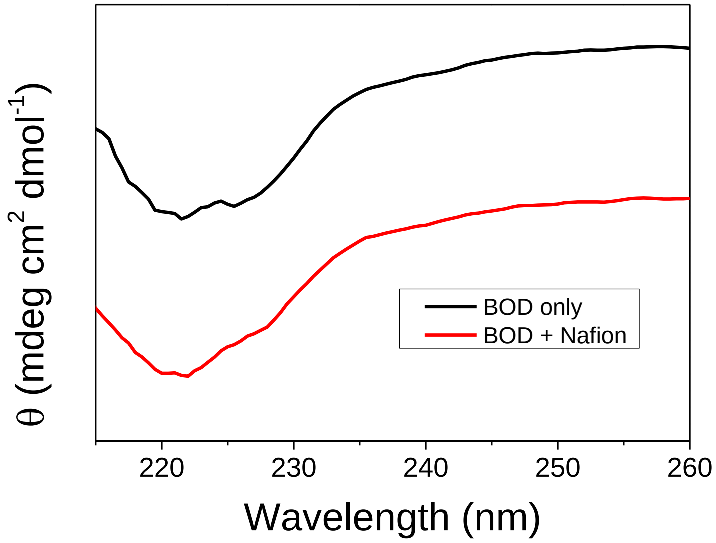

Supplementary Figure 2. Probing the secondary structure of the enzyme upon interaction with Nafion. Circular dichroism spectra of (●) BOD solution and (●) BOD + Nafion. Spectra were measured in a phosphate buffered electrolyte environment, pH 7.2.

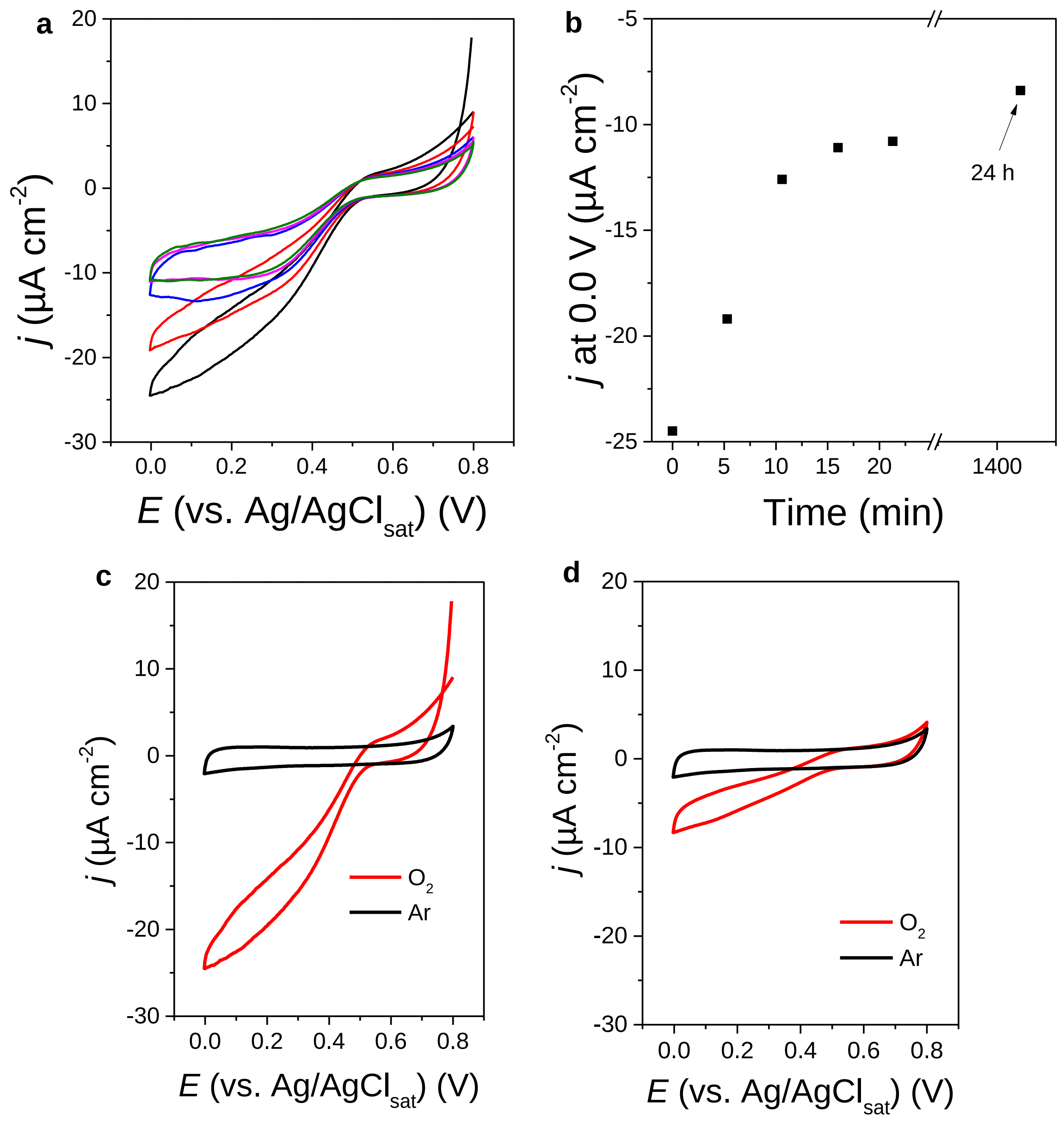

Supplementary Figure 3. Stability of the electrode for ORR. **a** Consecutive cyclic voltammograms of the *Mv*BOD-modified CCo electrode. **b** Loss of catalytic current along time. **c** First cyclic voltammogram and **d** after 24 h draining current. Supporting electrolyte: 0.1 mol L^{-1} phosphate buffer (pH 7.2), T = 25 °C. Scan rate: 5 mV s^{-1}. The electrode surface seems to stabilize itself after 4 voltammetric cycles, indicating a loss of 52% after the first 4 cycles.

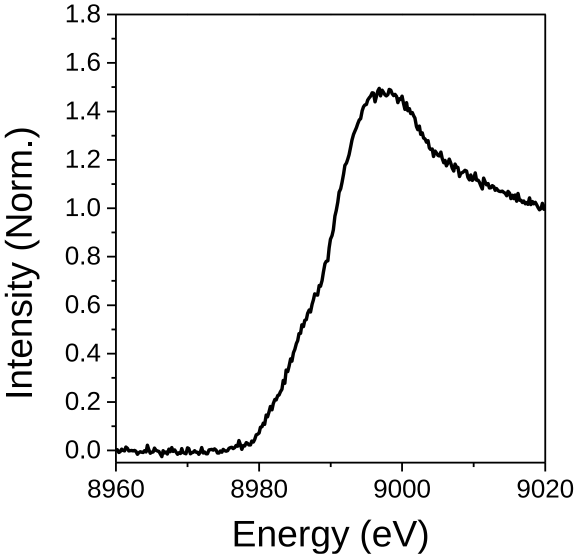

Supplementary Figure 4. Cu K-edge XAS spectrum of the *Mv*BOD-modified carbon electrode at OCP.

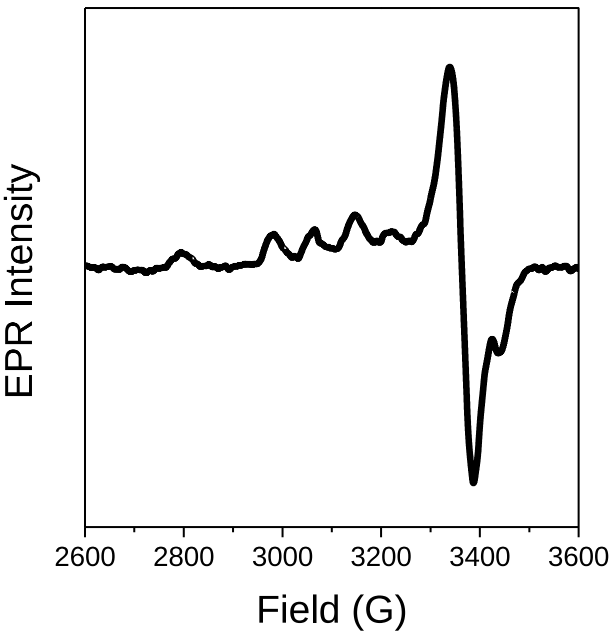

Supplementary Figure 5. X-band EPR spectrum of *Mv*BOD in phosphate buffer 0.1 mol L^{-1} (pH 7.2). T = 77 K. Acquisition parameters were set as follows: Power = 3 mW, Modulation amplitude = 3 G, Modulation frequency = 100 kHz, Time constant = 327.68 ms, Conversion time = 81.92 ms.

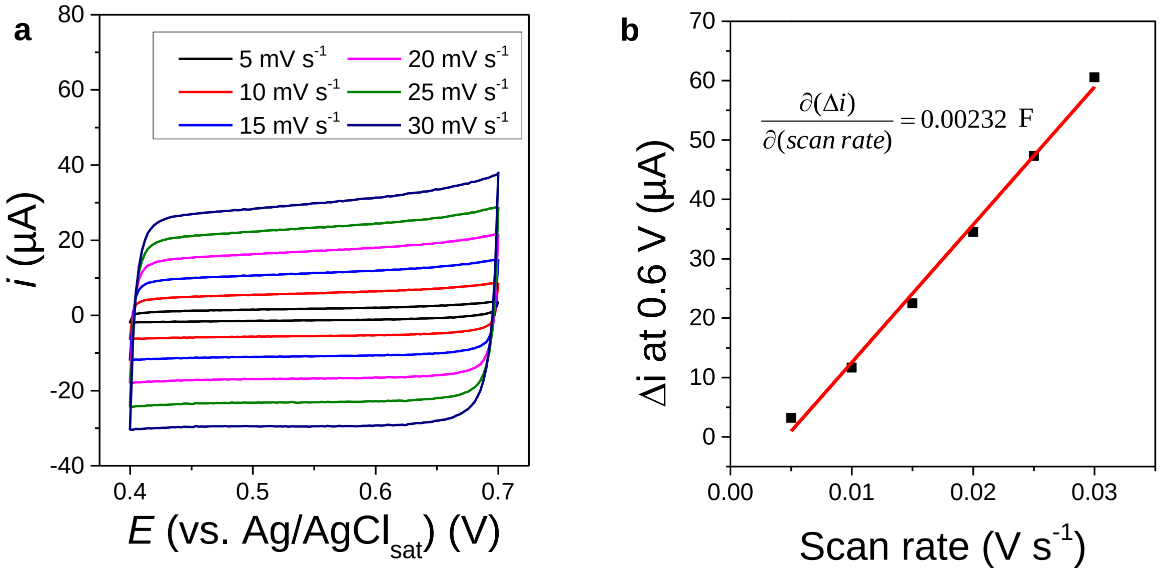

Supplementary Figure 6. Determining the electrochemical active surface area. **a** Cyclic voltammograms of the carbon electrode under different scan rates. **b** Difference of the capacitive currents at 0.6 V as function of the scan rate. Supporting electrolyte: phosphate buffer 0.1 mol L^{-1} (pH 7.2), T = 25 °C.

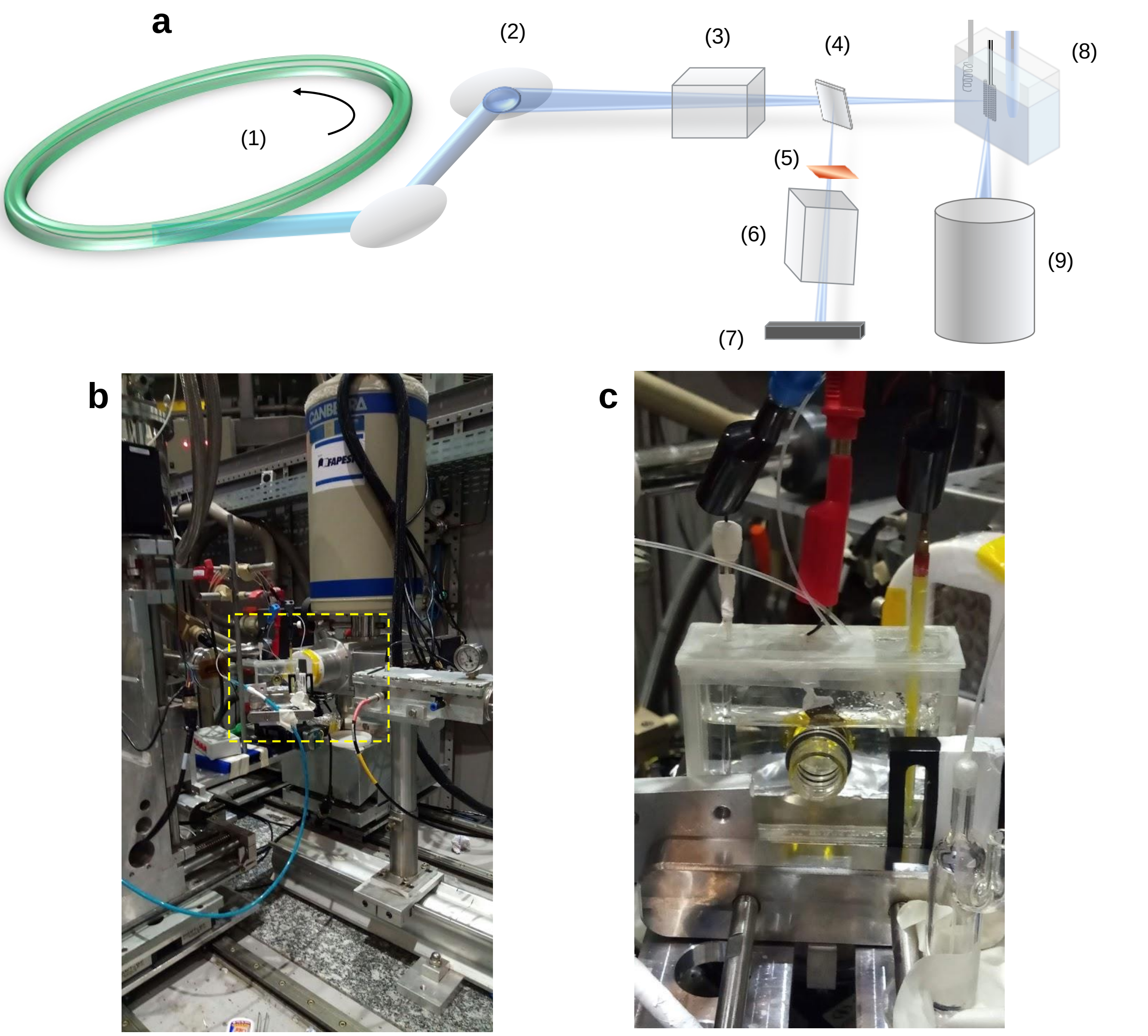

Supplementary Figure 7. XAS spectroelectrochemistry. **a** Experimental setup used in the *operando* XAS spectroelectrochemistry at the XAFS2 beamline of LNLS (photon flux 10^9 photons/s): (1) Storage ring, (2) Monochromator, (3) First ionization chamber (4) Beamsplitter, (5) Copper foil used as reference, (6) Second ionization chamber, (7) Beam blocker, (8) Electrochemical cell/Sample-holder, (9) Detector. **b** *In situ* XAS experimental arrangement, highlighting the home-made electrochemical cell. **c** Zoomed photograph of the electrochemical cell.

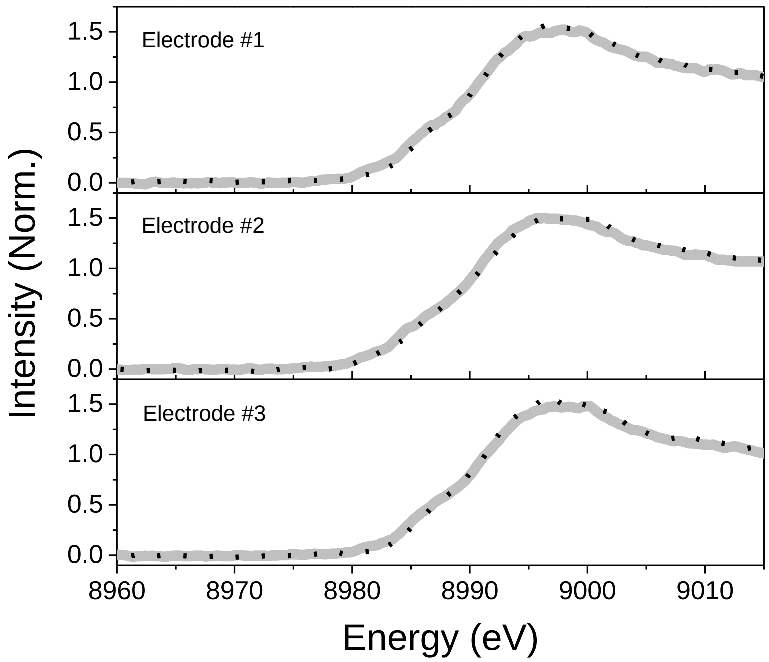

Supplementary Figure 8. Stability of the *Mv*BOD structure upon irradiation with x-rays. Spectra collected at the (continuous gray line) first measurements and (black dotted line) after 6 hours of operation in the spectroelectrochemical measurements.
